# Evaluation of a Global Initiative for Asthma Education and Implementation Program to Improve Asthma Care Quality (CARE4ALL): Protocol for a Multicenter, Single-Arm Study

**DOI:** 10.2196/65197

**Published:** 2025-01-08

**Authors:** Kewu Huang, Wenjun Wang, Ying Wang, Yanming Li, Xiaokai Feng, Huahao Shen, Chen Wang

**Affiliations:** 1 Department of Pulmonary and Critical Care Medicine, Beijing Chao-Yang Hospital, Capital Medical University Beijing Institute of Respiratory Medicine Beijing China; 2 Department of Pulmonary and Critical Care Medicine Beijing Hospital Beijing China; 3 Department of Pulmonary and Critical Care Medicine The Second Affiliated Hospital of Zhejiang University School of Medicine Hangzhou China; 4 National Center for Respiratory Medicine; State Key Laboratory of Respiratory Health and Multimorbidity; National Clinical Research Center for Respiratory Diseases; Institute of Respiratory Medicine Chinese Academy of Medical Sciences; Department of Pulmonary and Critical Care Medicine, Center of Respiratory Medicine China-Japan Friendship Hospital Beijing China; 5 Chinese Academy of Medical Sciences and Peking Union Medical College Beijing China

**Keywords:** asthma, management, quality improvement program, Global Initiative for Asthma, GINA, guidelines, implementation, health care, delivery

## Abstract

**Background:**

Poor symptom control and exacerbations of asthma diminish quality of life and pose a significant burden to patients and society. Implementing evidence-based management as recommended by the Global Initiative for Asthma (GINA), especially introducing inhaled corticosteroid–containing treatments, has the potential to vastly reduce exacerbations and the high burden of asthma in China. However, domestic implementation of the GINA recommendations has been unsatisfactory, especially in lower-level hospitals; thus, an enhancement to the awareness of and adherence to the GINA recommendations among Chinese physicians is needed to improve patient outcomes.

**Objective:**

This study aims to bridge the gap between the GINA recommendations and the current clinical practice in China by demonstrating the benefits of an asthma quality improvement program (QIP).

**Methods:**

A single-arm study will be conducted at around 30 hospitals across China to assess the impact of a specially designed asthma QIP. Approximately 1500 patients with asthma aged ≥14 years will be enrolled in participating hospitals and followed up for 48 weeks. The QIP—targeted at all pulmonologists and specialist nurses—will include an initial comprehensive training (including a pretraining questionnaire and posttraining quizzes) provided by a dedicated, qualified training team based on the GINA 2021 recommendations, followed by regular reinforcement learnings (integrated into the regular department lectures delivered by department directors), with multiple offline and online approaches (eg, an online patient management platform) provided as supportive tools. During this study, GINA implementation performance will be continuously monitored to inform necessary adjustments at the hospital level. The primary end point is change from baseline in the proportion of participants with an inhaled corticosteroid–based maintenance or reliever treatment at week 48. Secondary end points and exploratory end points include changes in clinical practice and patient outcomes such as treatment patterns, asthma control, and hospitalization rates due to exacerbations.

**Results:**

This study has been completed, with 1500 patients enrolled and 1271 patients completing the study. The last visit of the last patient was on September 3, 2024, and the database lock was on September 28, 2024. Final analysis of data has started in October 2024.

**Conclusions:**

The Change Asthma Clinical Practice through GINA Education and Implementation for All Patients With Asthma (CARE4ALL) study will hopefully help improve asthma management and patient outcomes in China by bridging the gap between evidence-based GINA recommendations and the current clinical practice.

**Trial Registration:**

ClinicalTrials.gov NCT05440097; https://clinicaltrials.gov/study/NCT05440097

**International Registered Report Identifier (IRRID):**

DERR1-10.2196/65197

## Introduction

Asthma is a chronic inflammatory disease, characterized by repeated episodes of breathlessness, wheezing, chest tightness, and coughing, which affects more than 330 million people worldwide [1]. Within China, as reported by the China Pulmonary Health study, the prevalence of asthma is 4.2% among adults aged at least 20 years, representing 45.7 million patients, and it is anticipated to increase further due to changes in environment and lifestyle [2]. However, the current status of asthma management in China is far from satisfactory.

Asthma control is defined as the level to which the various manifestations of asthma have been reduced or eliminated by treatment [3]. As it is closely linked to patients’ health status, the achievement of good asthma control has been set as the key element that drives patient management [3-6]. The rate of well-controlled asthma, as defined by the Global Initiative for Asthma (GINA), is only 28.5% in China’s urban areas [7] and is expected to be even lower in remote areas [8]. Moreover, poor asthma control is associated with a much higher risk of exacerbations, which is a major cause of disease morbidity and medical resource use [9]. According to a multinational, cross-sectional survey, 17.8% of patients with asthma in China experienced at least one exacerbation within the past 12 months [10], much higher than 8.4% in the United Kingdom and 12.5% in the United States [11]. Previous studies revealed that the suboptimal asthma control and the high exacerbation burden in China might be largely attributed to underdiagnosis and undertreatment in asthma management [2,10,12]. Though the underlying reasons may be complicated, low awareness of and adherence to guideline recommendations among physicians, insufficient disease awareness, and poor treatment adherence (including incorrect technique of inhalers) among patients might all play a part [12,13].

The GINA strategy report (also known as GINA) has been updated annually since 2002 to provide physicians with up-to-date, evidence-based recommendations for asthma prevention and management [6]. Commencing asthma treatment with short-acting β_2_-agonists (SABAs) alone has been a long-standing approach in the field [14], but concerns were raised as overuse of SABAs was shown to be associated with an increased risk of asthma-related death [15]. As more relevant evidence emerged, in 2019, GINA concluded that adults and adolescents with asthma should not be treated with SABAs alone for consideration of safety, regardless of asthma severity [14]. Since then, inhaled corticosteroid (ICS)–containing therapies have been recommended for all patients with asthma by both international and Chinese guidelines [6,8,14]. However, ICS-containing therapies are largely underused in China. For example, in a multinational cross-sectional survey conducted in 2020 on physicians (including general practitioners, family medicine physicians, or internal medicine physicians), the results from China showed that 31.9% of the respondents regarded inhaled SABAs only as the typical treatment of mild asthma, suggesting the suboptimal awareness of guideline recommendations among Chinese physicians [16]. Considering the critical role that physicians play in the delivery of asthma care, limited understanding and implementation of guideline recommendations would inevitably translate to compromised patient outcomes.

The unsatisfactory quality of medical care and the high disease burden call for national actions to increase physicians’ awareness of and adherence to management recommendations to improve patient outcomes [17]. Studies from China have shown that interventions targeted by health care professionals at the hospital level can increase physicians’ adherence to guideline recommendations and, in turn, improve patient asthma outcomes [18,19]. However, the existing studies either only conducted a short-term, one-off intervention program [18] or were limited to a single study center [19]. Such interventions can be better delivered in the form of a quality improvement program (QIP), which is a set of systematic and continuous activities designed to monitor, analyze, and improve the quality of health care processes [20]. QIPs for asthma care have been demonstrated to effectively change physician practices and improve clinical outcomes in other countries [21]. For example, the Enhancing Care for Patients With Asthma study, developed to augment the implementation of the Expert Panel Report 3 Guidelines in four American states [22], successfully improved the consistency of practices with the Expert Panel Report 3 Guidelines and subsequently decreased asthma-related emergency department visits and hospitalizations by 37.7% and 47.1%, respectively, based on a retrospective analysis [23]. QIPs have also been successfully conducted in China for other diseases [24,25], but the feasibility and benefits of such QIPs for asthma care have not been assessed in China.

The Change Asthma Clinical Practice through GINA Education and Implementation for All Patients With Asthma (CARE4ALL) study will conduct the first-ever multifaceted QIP for China asthma care, targeting physicians with a specialization in pulmonary or respiratory care. The QIP aims to bridge the gap between the recommendations from the GINA 2021 (the latest update at the time of study design) and the clinical practice among participating health care professionals, and to improve patient outcomes through enhanced quality of care. By evaluating the clinical impact of such a QIP in a nationwide cohort, the ultimate objective of the CARE4ALL study is to pilot and establish a widely applicable QIP model for improving domestic asthma management in China.

## Methods

### Study Design

**Figure 1:**
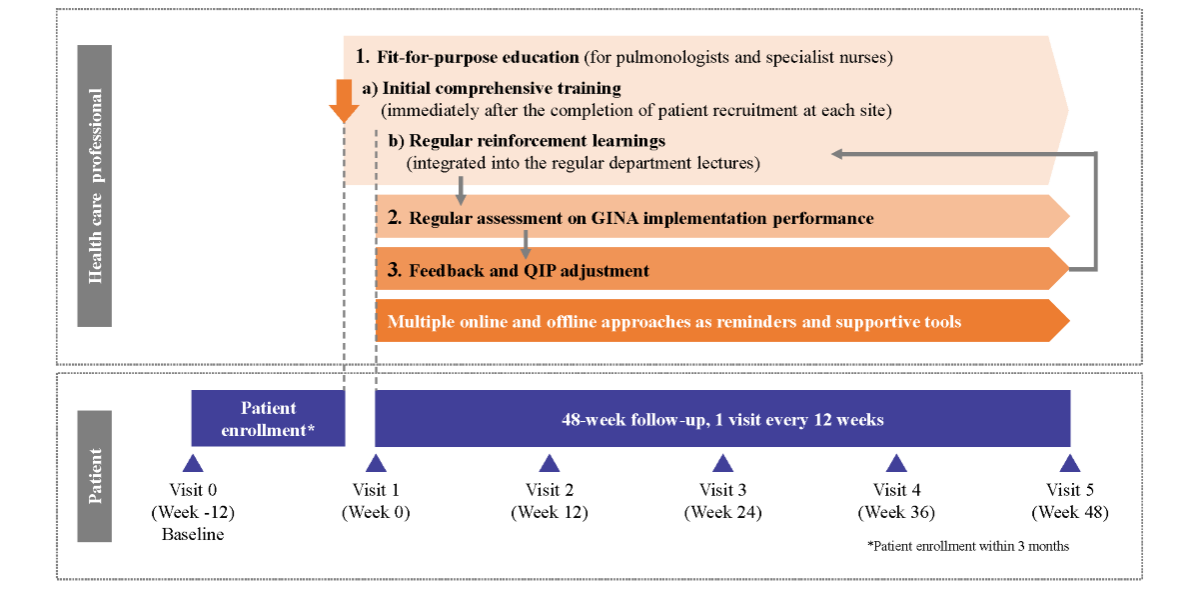
Study design. GINA: Global Initiative for Asthma; QIP: quality improvement program.

This is a multicenter, single-arm study (NCT05440097) with primary data collection to assess the impact of an asthma QIP in improving the adherence of health care professionals specializing in pulmonary or respiratory care to recommendations from the GINA 2021 and quality of care. This study’s methodology and procedures are shown in Figure 1.

### Study Sites and Participants

#### Site Selection

This study selected a total of around 30 tertiary and secondary hospitals that met all the following criteria: (1) classified as public general hospitals (excluding traditional Chinese medicine hospitals), (2) having an emergency unit and a respiratory department with wards and fundamental equipment (eg, spirometry), (3) having access to GINA-recommended treatment regimens, (4) visited by ≥500 patients with asthma in the past one year, and (5) willing to provide all pulmonologists with regular GINA education and comply with GINA 2021 recommendations. To represent the real-world asthma care situation in mainland China, hospitals were selected across as many provinces or municipalities as possible located below an altitude of 1500 meters, with a balance of hospital levels (preferably around 20 at the tertiary level and around 10 at the secondary level).

#### Patients

Outpatients with physician-confirmed asthma will be consecutively recruited at participating hospitals. Patients will be eligible for enrollment if they are ≥14 years old and provide written informed consent. Exclusion criteria include previous diagnosis of clinically relevant chronic respiratory disease other than asthma (eg, chronic obstructive pulmonary disease); any significant medical conditions that may put the patient at risk, influence this study’s results, or hinder the participants from fully complying with this study’s procedure; any medical conditions other than asthma that requires treatment with systemic or oral steroids; and participation in another clinical study with an investigational product administered in the last three months before visit 1 (Figure 1).

### Study End Points

As the QIP aims to align physicians’ clinical practice with GINA recommendations, study end points measured to what extent the physicians’ practice complied with GINA-recommended best practice, which could be reflected in various aspects. First, patient medication patterns directly reflected whether physicians had prescribed asthma medications as recommended by GINA. Thus, the primary end point measured the change from baseline at week 48 in the proportion of patients receiving ICS-based maintenance or reliever medications (as the mainstay of the GINA-recommended asthma medication scheme), while several secondary end points were designed to characterize more fully the patients’ asthma medication patterns (Textbox 1). Second, the physician’s knowledge, skill, and action together reflected their understanding of and ability to implement GINA strategies. Thus, the exploratory end points included measurements on the proportions of patients whose pulmonologists develop or review an asthma action plan (a GINA-recommended management tool) for them and check to ascertain their inhaler technique, as well as scores measuring the physicians’ own asthma knowledge and inhaler technique (Textbox 1). Moreover, the physicians’ patient management skills and the quality of their communication and education for the patients were expected to improve through the QIP, which in turn would positively influence the patients’ disease knowledge levels and self-management behavior. As such, the exploratory end points included assessments of the patients’ asthma knowledge, inhaler skills, and compliance, to serve as indirect indicators of the physicians’ patient management proficiency (Textbox 1).

By aligning current clinical practice with GINA recommendations, the ultimate goal of the QIP is to enhance patient outcomes, as improved quality of care is expected to bring about better asthma management results. Therefore, several key secondary end points sought to evaluate changes from baseline in patients’ asthma control (assessed by the five-item Asthma Control Questionnaire), and patients’ health-related quality of life and incidence of severe asthma exacerbation were also evaluated as exploratory end points (Textbox 1). Together, these end points on patient outcomes will help demonstrate the clinical benefits that can be actualized through the QIP.

**Study end points and outcome measures. Asthma treatment at each study visit refers to the treatment received within the prior 12 weeks according to medical records. For instance, in the primary end point, “a participant with an ICS-based maintenance or reliever therapy at week 48” is defined as a participant who has used inhaled corticosteroid (ICS)–based maintenance or reliever therapy between week 36 and week 48 based on medical records and in-hospital or out-of-hospital prescriptions.**

**Primary**

- Change from baseline in the proportion of participants with an ICS-based maintenance or reliever therapy at week 48.

**Secondary**

- Change from baseline in the proportion of participants with well-controlled asthma (five-item Asthma Control Questionnaire [ACQ-5] ≤0.75) at week 48.
- Distribution of ACQ-5 scores [proportion of participants with well-controlled (ACQ-5 ≤0.75), partially controlled (0.75<ACQ-5≤1.5), and not well-controlled (ACQ-5 >1.5) asthma] at weeks 12, 24, 36, and 48.
- Change from baseline in the proportion of participants on the treatment of ICS-formoterol as a reliever at weeks 12, 24, 36, and 48.
- Change from baseline in mean ACQ-5 scores at weeks 12, 24, 36, and 48.
- Change from baseline in the proportion of participants achieving an improvement in ACQ-5 of ≥0.5 units at weeks 12, 24, 36, and 48.
- Change from baseline in the proportion of participants with an ICS-based maintenance or reliever treatment at weeks 12, 24, and 36.
- Distribution of asthma treatment (eg, ICS-containing medications, ICS-long-acting β_2_-agonist, ICS-formoterol, oral corticosteroids, leukotriene receptor antagonists, theophylline, and traditional Chinese medicine) at baseline and weeks 12, 24, 36, and 48.

**Exploratory**

- The proportion of participants whose pulmonologists developed or reviewed the written asthma action plan at weeks 0, 12, 24, 36, and 48.
- The proportion of participants whose pulmonologists watched the patient using their inhaler to check their technique at weeks 0, 12, 24, 36, and 48.
- Annual hospitalization rate due to asthma exacerbations per patient.
- Number of severe asthma exacerbations at baseline, weeks 12, 24, 36, and 48.
- Change from baseline in health-related quality of life evaluated by mean Standardized Asthma Quality of Life Questionnaire for 12 years and older scores at weeks 0, 12, 24, 36, and 48.
- Change from baseline in the proportion of participants with an ICS-based maintenance or reliever actual treatment at week 48.
- Change from baseline in mean Medication Adherence Report Scale for Asthma scores at weeks 12, 24, and 48.
- Change from baseline in the inhaler skill scores of pulmonologists at weeks 12, 24, and 48.
- Change from baseline in the inhaler skill score of patients at weeks 12, 24, and 48.
- Change from baseline in the scores of the asthma knowledge questionnaire for patients at weeks 12, 24, and 48.
- Change from baseline in the scores of the patient expectation of asthma treatment questionnaire at weeks 12, 24, and 48.
- Change from baseline in the scores of the asthma knowledge questionnaire for pulmonologists at weeks 12, 24, and 48.
- Level of asthma control at baseline by self-assessment and by the Global Initiative for Asthma assessment.
- Level of asthma control at baseline, weeks 0, 12, 24, 36, and 48.
- Patient characteristics and related symptoms after COVID-19 infection.

### Interventions

**Figure 2:**
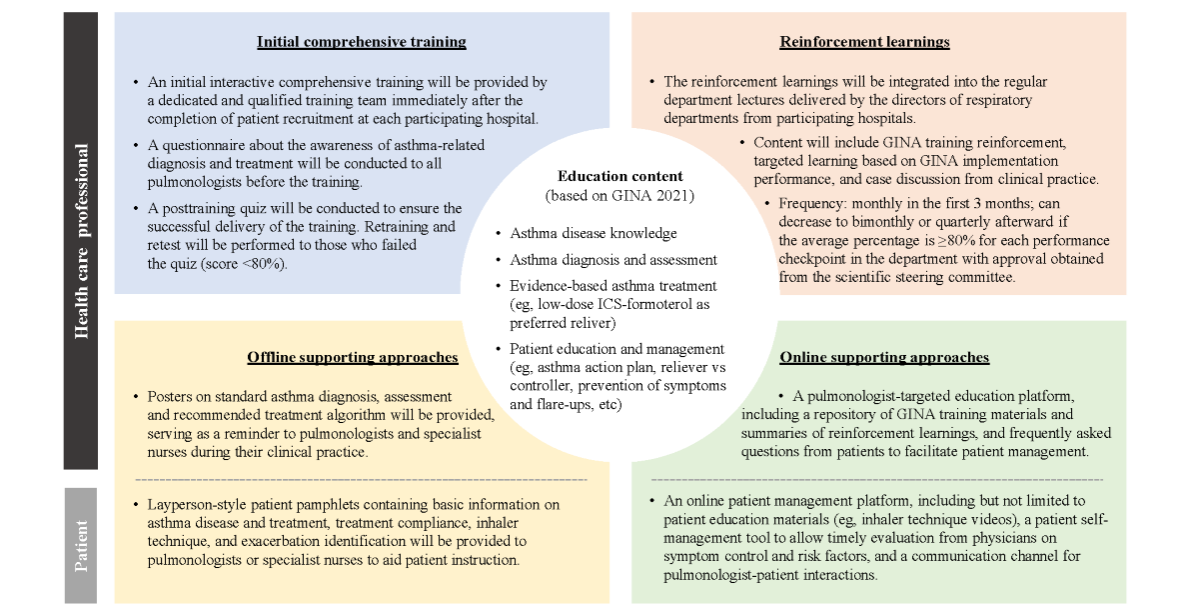
Multifaceted approaches designed to facilitate GINA implementation in the QIP. GINA: Global Initiative for Asthma; ICS: inhaled corticosteroid; QIP: quality improvement program.

The QIP delivered at the hospital level targeted all pulmonologists and specialist nurses at each participating hospital with the goal of encouraging asthma management per the GINA 2021 recommendations in their routine clinical practice. The program included initial comprehensive training, which was delivered by a dedicated qualified team (including asthma experts in the same province or region as the participating hospital) immediately after the completion of patient recruitment at each site. This timing of training commencement was designed to avoid biasing the baseline data of clinical characteristics by training-induced behavior changes. The education content was based on recommendations from the GINA 2021, including, but not limited to, asthma diagnosis and assessment, evidence-based asthma treatment and the scientific rationale, and patient education and management (eg, asthma action plan, the rationale for medication adherence, etc). A posttraining quiz was conducted to ensure the successful delivery of the training. Thereafter, pulmonologists and specialist nurses were required to attend regular reinforcement learnings to enhance their understanding of GINA-recommended asthma management and address practice gaps identified from the GINA implementation performance assessment. During this study’s period, multiple online and offline approaches served as supportive tools to increase adherence to the GINA recommendations (Figure 2). In addition to pulmonologists- or nurses-targeted efforts, layperson-style patient pamphlets and an online patient management platform were developed to help enhance patients’ adherence to asthma treatment (Figure 2). The platform housed patient education materials, incorporated a patient self-management tool, and provided a communication channel to facilitate physician-patient interactions.

A scientific steering committee consisting of external asthma experts, most of whom are committee members of the Asthma Group of the Chinese Thoracic Society, was established to control the overall quality of the QIP. The committee reviewed and approved the overall design of this study and ensured that the education plan and related materials were scientifically and clinically appropriate. The committee was also involved in the review and interpretation of this study’s results.

Monthly assessments of GINA implementation performance were conducted to guide QIP adjustments (eg, targeted reinforcement learnings) at the hospital level. GINA implementation performance was assessed against 6 predefined key performance indicators (Textbox 2) for each participant’s visit. The proportion of participants achieving each checkpoint was calculated at the hospital level, which can help identify gaps in GINA implementation and determine future targeted training needs. Assessment feedback was provided to the scientific steering committee and the directors of participating departments. If the GINA implementation performance was unsatisfactory at a participating department (defined as the average percentage at the department level lower than 80% for at least one performance checkpoint), the scientific steering committee discussed the root cause with the department director and provided instructions for an improved plan.

**Key performance indicators for Global Initiative for Asthma implementation performance and data source.**

**Questionnaires collected from participating patients after each visit**

- Whether or not the pulmonologist or the specialist nurse assessed the patient’s symptom control over the past four weeks?
- Whether or not the pulmonologist or the specialist nurse watched the patient using their inhaler?
- Whether or not the pulmonologist discussed treatment adherence?
- Whether or not the pulmonologist developed or reviewed the written asthma action plan?
- Whether or not the pulmonologist reduced the dosage of asthma treatment? If yes, whether the patient had a pulmonary function test before dosage reduction?

**Medical records**

- Whether or not the pulmonologist has provided ICS-containing medication for maintenance or as a reliever?

### Assessment and Data Collection

The electronic data capture system was used for data collection and query handling, and all data was recorded in the electronic case report form per prespecified instructions. All data were obtained from medical records generated during routine clinical practice except for the questionnaires to be completed by patients. During this study, usual care activities were performed as needed and no additional examinations were required.

At each study visit (including baseline and follow-up visits, Figure 1), medical records on asthma-related assessment and treatment during the past 12 weeks were collected, and two patient-reported outcome questionnaires—a five-item Asthma Control Questionnaire and Standardized Asthma Quality of Life Questionnaire—for patients aged 12 years and older was administered. The Medication Adherence Report Scale for Asthma, the asthma knowledge questionnaire for patients (study-defined, designed based on The Validity and Reliability of an Asthma Knowledge Questionnaire Used in the Evaluation of a Group Asthma Education Self-Management Program for Adults With Asthma), the patient inhaler skill assessment and the patient expectation of asthma treatment questionnaire (study-defined) will also be administered at baseline or follow-up visits (Multimedia Appendix 1). Additionally, patients’ demographics, clinical characteristics within three months, and historical data (eg, asthma history, comorbidities, and comedication history) were collected at baseline, and hospitalization and outpatient visit records related to asthma were collected at follow-up visits (Multimedia Appendix 1). To better reflect real-world conditions, no free medications were provided in this study, and information on the sources from which patients purchase their medications was collected at baseline. Participants were encouraged to return to this study’s hospital if they had asthma-related conditions or worsening symptoms. In case of emergency, they could choose to visit a hospital other than this study’s hospital but were required to report it to study pulmonologists or study staff with supporting documents (eg, medical record of hospitalization summary). Data generated from other hospitals were collected as per protocol. A patient might withdraw from this study at any time at their own request; at the time of withdrawal from this study, an early study discontinuation visit was conducted for any data that needed to be collected.

### Data Analysis

#### Sample Size Estimation

With a 30% dropout rate and a within-participants correlation of 0.25, approximately 1500 patients are required to provide an 80% power at a significant level of .05 to detect an increase from baseline of 5% at week 48 in the proportion of patients with an ICS-based maintenance or reliever therapy, which is assumed to be 40% at baseline [26].

#### Statistical Plans

The main analyses that assess the impact of QIP were performed using the full analysis set (FAS), which consisted of all enrolled participants with at least one nonmissing postintervention GINA treatment assessment. Baseline demographics and characteristics will be presented for all enrolled participants and FAS. Continuous variables will be summarized descriptively as appropriate. Categorical variables will be presented as frequency counts and percentages. When applicable, 95% CIs will be presented with estimates of proportions.

Analysis of the primary end point were conducted in the FAS with a mixed effect logistic regression model, considering the measurement time point (baseline or post baseline) as the fixed effect and hospital, pulmonologist, and patient as the random effects. In case of lack of convergency, the hospital and pulmonologist were removed from the model. A sensitivity analysis was conducted with a generalized estimating equations model including the same covariates as in the primary analysis. Secondary and exploratory end points were presented primarily with summary statistics. For the analysis of end points, by-visit end points were analyzed using observed data, and missing data were not imputed.

Subgroup analyses were performed as appropriate according to age (<18, 18–65, or >65 years), age of asthma onset (<20, <40, or ≥40 years), phenotype of asthma (allergic or nonallergic), hospital level (tertiary or secondary), occupation (asthma-related or not), baseline characteristic, questionnaire response status (with or without response), adherence, patient expectation of asthma treatment, asthma history, asthma severity class, type of the participating hospital department (pulmonary and critical care medicine or nonpulmonary and critical care medicine), geographic region (North or South), change from baseline in ICS-containing treatment (with-to-with, with-to-without, without-to-with, or without-to-without ICS-containing treatment), and change from baseline in ICS-formoterol as reliever (with-to-with, with-to-without, without-to-with, or without-to-without ICS-formoterol as reliever). Any participants with a missing value for a predefined subgroup were excluded from the analysis of that subgroup.

### Ethical Considerations

This study’s protocol had been approved by the Ethics Committee of Beijing Chao-Yang Hospital, Capital Medical University (2022-KE-22).

Informed consent was obtained from a participant or their legally authorized representative before conducting any procedure specifically for this study on the participant.

Participants were assigned a unique identifier by the sponsor. Any participant records or datasets that were transferred to the sponsor contained the identifier only; participant names or any information that would make the participant identifiable were not transferred.

Participant payment outlines were discussed in the informed consent process. Participants were paid a CN ¥ 200 (approximately US $27.50 as of December 12, 2024) transportation fee per on-site visit for reasonable expenses incurred due to their participation in this study.

## Results

This study has been completed, with 1500 patients enrolled and 1271 patients completing the study. The last visit of the last patient was on September 3, 2024, and the database lock was on September 28, 2024. Final analysis of data has started in October 2024.

## Discussion

### Rationale and Study Design

Poor symptom control and exacerbations of asthma impair work and activities, diminish quality of life, and pose a significant burden to patients and society [9,27]. Implementing evidence-based management as recommended by GINA has the potential to improve the quality of domestic asthma care and vastly reduce the disease burden of asthma in China [17]. In line with GINA recommendations, the latest versions of the Chinese guidelines and an expert consensus for asthma management also advocate for ICS-based maintenance and relieving treatment [8,28]. Meanwhile, ICS-containing medications such as budesonide and budesonide-formoterol have been included in China’s National Essential Drug List and National Reimbursement Drug List, ensuring patients’ access to these asthma medications (including those with special needs such as school-aged patients who may require more than one canister with a single prescription to maintain medication availability both at school and at home). However, at present, domestic asthma control is still unsatisfactory with patients’ treatment patterns deviating from the GINA recommendations, suggesting that Chinese physicians’ suboptimal awareness of and adherence to the recommendations [2,12,16] may be an important underlying factor that warrants further improvement. Experience from other countries has demonstrated that clinical benefits can be achieved through QIPs for asthma care [21-23]. As such, the CARE4ALL study will conduct China’s first-ever nationwide multifaceted QIP targeting physicians with a specialization in pulmonary or respiratory care, which aims to transform the suboptimal asthma control status in China by bridging the gap between GINA recommendations and current clinical practice among Chinese physicians.

### Expected Results

We expect that the physicians’ awareness of and adherence to the GINA recommendations will be enhanced through the QIP, which would be manifested as more widespread clinical practices that are per evidence-based asthma management, including the implementation of standard, GINA-recommended treatment. We further anticipate that the improved quality of care provided by the physicians would translate to better asthma control and health-related quality of life among the patients. If this study demonstrates these benefits of the QIP in standardizing asthma management in China, this QIP could be considered as a standard model that the whole country can apply to reduce the burden of asthma in China.

### Strengths

As the first nationwide QIP for asthma care with a prospective evaluation of clinical impacts in China, the CARE4ALL study has several strengths in its design which differentiate it from previous simple, small-scale educational interventions. First, multifaceted intervention will be provided for physicians to aid understanding and encourage close adherence to the GINA recommendations. The initial comprehensive training intends to familiarize physicians with GINA, which will be reinforced by regular learning sessions typically unseen in routine educational events. Additionally, both online and offline supporting materials are readily available to physicians for easy reference and patient education. Second, different from routine physician-oriented educational events and toolbox, this QIP will also provide physicians with support to facilitate patient management in clinical practice. As effective asthma care requires patients to be actively engaged in multiple self-management behaviors, improving patients’ adherence with GINA-recommended practices (eg, ICS-containing medications, appropriate device technique, use of an asthma action plan, etc) is of great importance to the ultimate success of the GINA implementation. Notably, apart from layperson-style patient education materials aiming to raise patients’ awareness of disease management and treatment strategies, an online patient management platform will be developed to facilitate timely evaluations of symptoms and effective physician-patient communications. These interventions are expected to improve patients’ treatment adherence and consequently asthma control as demonstrated by previous studies [29,30].

Third, the GINA implementation at the hospital level will be monitored based on the 6 performance indicators and dynamically improved based on feedback from the scientific steering committee, which consists of national asthma experts. Therefore, all the participating hospitals can benefit from constructive instructions from the committee. Finally, while primary health care facilities in remote rural areas will be excluded due to their inadequate administrative and data management capacity, this study will encompass both tertiary and secondary hospitals from as many provinces or municipalities as possible, aiming to provide unprecedented coverage to better represent the real-world situations in China [18,19]. In China’s current medical system, most asthma patients are diagnosed and treated under specialist care rather than by general practitioners, thus this study, by specifically targeting pulmonologists, will exert a direct and substantial effect on shaping the clinical practice of asthma care in China’s medical system. As such, experiences and lessons from the program will hopefully inform a valuable model for asthma care improvement in the whole of China as well as for similar programs for other chronic diseases.

This QIP is expected to exert a sustained impact on asthma care practices. First, unlike brief educational events [18], the intervention for our study will last for one year, providing a sustained framework for cultivating evidence-based clinical practice behaviors in physicians. Second, both online and offline supporting materials used during the QIP will continue to be accessible to physicians after the program finishes; coupled with continuous assessments of GINA implementation performance, these resources will aid in maintaining physicians’ awareness and adherence to GINA-recommended asthma management. Lastly, by not providing free medications, this study will closely mirror any potential influence that medication accessibility or affordability may have on patients in real-world conditions, thereby enhancing the generalizability of the findings.

### Limitations

This study has two key limitations. First, it lacks a concurrent control arm. Instead, it follows the before-and-after design and compares variables measured before and after the QIP, which is an approach often adopted in QIP-evaluating studies for the purposes of better reflecting real-world settings and maintaining simplicity. The effectiveness of this before-and-after design in reflecting the effects of QIP interventions has been widely demonstrated [23,31,32]. Second, multiple sources will be used for data collection, likely leading to missing and inconsistent data. Given that patients may be admitted to other hospitals due to exacerbations, data collection from multiple sources will be inevitable. Past and new hospitalizations out of the study hospital due to exacerbations will be recorded based on medical records rather than patients’ personal accounts to reduce missing data and minimize recall bias.

### Conclusions

In summary, the CARE4ALL study should help improve asthma management and patient outcomes in China by bridging the gap between evidence-based GINA recommendations and the current clinical practice. This multifaceted QIP is expected to provide valuable insights for further quality improvement in asthma care at the national level.

## Appendix

Multimedia Appendix 1 Protocol schedule of activities.

## Abbreviations

CARE4ALL: Change Asthma Clinical Practice through Global Initiative for Asthma Education and Implementation for All Patients With Asthma
FAS: full analysis set
GINA: Global Initiative for Asthma
ICS: inhaled corticosteroid
QIP: quality improvement program
SABA: short-acting β2-agonist

## Data Availability

The datasets generated or analyzed during this study will be available from the corresponding author upon reasonable request.
