# Supplementary material for: Evaluation of a Global Initiative for Asthma Education and Implementation Program to Improve Asthma Care Quality (CARE4ALL): Protocol for a Multicenter, Single-Arm Study
Source: JMIR Res Protoc. 2025 Jan 8;14:e65197. doi: 10.2196/65197 (PMC11754978; doi:10.2196/65197)
Supplement: Multimedia Appendix 1 [file resprot_v14i1e65197_app1.docx]

|  | **Baseline Period** | **Intervention  Period** | | | | |
| --- | --- | --- | --- | --- | --- | --- |
| **Study Visit** | **V0** | **V1** | **V2** | **V3** | **V4** | **V5  (Study completion/ withdrawal)** |
| Weeks | -12±1 | 0±2 | 12±1 | 24±1 | 36±1 | 48±1 |
| Informed consent ^a^ | X |  |  |  |  |  |
| Inclusion and exclusion criteria | X |  |  |  |  |  |
| Demographics ^b^ | X |  |  |  |  |  |
| Vital sign ^c^ | X |  |  |  |  |  |
| Participant status ^d^ | X |  |  |  |  |  |
| Smoking status ^e^ | X |  |  |  |  |  |
| Medical history (comorbidities) and medications ^f^ | X |  |  |  |  |  |
| Asthma history & related treatment ^g^ | X |  |  |  |  |  |
| Severe asthma exacerbation |  | X | X | X | X | X |
| Hospitalisation due to asthma exacerbation since last visit |  | X | X | X | X | X |
| ACQ-5 | X | X | X | X | X | X |
| AQLQ(S)+12 | X | X | X | X | X | X |
| PFT | X |  |  |  |  | X |
| MARS-A questionnaire | X |  | X | X |  | X |
| Asthma knowledge questionnaire for patients | X |  | X | X |  | X |
| Inhaler skill score for patients |  | X | X | X |  | X |
| Patient expectation of asthma treatment questionnaire | X |  | X | X |  | X |
| Laboratory assessments within prior 3 months (if available in medical records) ^h^ | X | X | X | X | X | X |
| PFT/BPT/PEF/FeNO within prior 3 months (if available in medical records) | X | X | X | X | X | X |
| Medical records on asthma related assessment and treatment | X | X | X | X | X | X |
| Questionnaire for Asthma Patients during COVID-19 ^i^ | X | | | | | |
| Patient evaluation on whose pulmonologists 1) assess symptom control, 2) watch the patient using their inhaler, check their technique, 3) discuss adherence, 4) develop or review the written asthma action plan, provide patient with education, 5) have a PFT before dosage reduction | X | X | X | X | X | X |

ACQ-5, five-item Asthma Control Questionnaire; AQLQ(S)+12, Standardised Asthma Quality of Life Questionnaire for 12 years and older; PFT, pulmonary function testing; BPT, bronchial provocation test; MARS-A, Medication Adherence Report Scale for Asthma; PEF, peak expiratory flow; FeNO, fractional exhaled nitric oxide.

a. Informed consent must be conducted prior to performing any study procedures including data collection.

b. Demographics include patient age, gender, race, ethnicity.

c. Vital signs include body mass index (BMI, calculation by height and weight)

d. Participant status include education level, family monthly income, job, residence (urban communities or rural villages), etc.

e. Smoking status include current smoker or stopping smoking or restarting smoking, smoking pack-years.

f. Asthma related comorbidities, including allergic history, rhinitis, chronic rhinosinusitis, gastroesophageal reflux disease, obesity, obstructive sleep apnoea, depression, anxiety, eczema, and atopic dermatitis according to ICD-10-CM. Medication includes drugs administered for these asthma related comorbidities within 4 weeks before enrolment.

g. Historical data, e.g., the first diagnosed date of asthma and total number of prescription of systemic glucocorticoid treatment for at least 3 days or emergency visit or hospitalisation due to asthma exacerbations during the previous 12 months (an emergency room visit due to asthma that required use of systemic corticosteroids or hospitalisation due to asthma), home PEF availability, asthma symptoms, severity class evaluated by self-assessment, asthma control level, first asthma-diagnose hospital and tier level, hospital numbers for asthma clinic visit in past 12 month, medical insurance category, patient’s expectation for asthma treatment, etc.

h. Clinical laboratory testing data include complete blood count with differentiation; blood gas analysis (pH, SaO2, PaO2, PaCO2, and HCO3-); C-reactive protein; skin allergen prick, total IgE or specific IgE.

i. Questionnaire for Asthma Patients during COVID-19 is required exclusively for patients infected with COVID-19; these patients are required to answer the questionnaire anew with each infection.
